# Influence of theobromine in feed on larval growth and survival in *Tenebrio molitor*

**DOI:** 10.1371/journal.pone.0328354

**Published:** 2025-07-15

**Authors:** Matthieu Bourdarias, Sofie Landschoot, Mia Eeckhout, Myriam Hesta

**Affiliations:** 1 Department of Food Technology, Faculty of Bioscience Engineering, Ghent University, Gent, Belgium; 2 Department of Plant and Crops, Faculty of Bioscience Engineering, Ghent University, Gent, Belgium; 3 Department of Morphology, Imaging, Orthopedics, Rehabilitation and Nutrition, Faculty of Veterinary Medicine, Ghent University, Gent, Belgium; University of Tennessee, UNITED STATES OF AMERICA

## Abstract

With the growing interest in using insects as a sustainable protein source for human and animal consumption, identifying suitable feedstuffs is crucial for their rearing. Although *Tenebrio molitor* larvae can valorise food industry byproducts, the presence of certain compounds may limit this potential. This study assesses the impact of theobromine, a methylxanthine found in chocolate, on the growth and mortality of *Tenebrio molitor* larvae. The aim was to evaluate the feasibility of rearing mealworms on byproducts and former foodstuffs from the biscuit, confectionery and chocolate industries. Larvae were fed varying concentrations of theobromine (up to 5000 mg/kg) in their diet under controlled laboratory conditions. Weight and mortality were monitored until 10% pupation was observed. Results indicated that concentrations up to 600 mg/kg of theobromine had no significant effect on overall larval growth. Concentrations up to 1600 mg/kg did not significantly affect final weight but caused weight differences during growth, and only exposure to 5000 mg/kg appeared to increase mortality. These findings suggest that exceeding a threshold of theobromine influences larval growth, and high exposure could lead to increased mortality. This study expands the potential range of byproducts, including those containing chocolate, that could be incorporated into mealworm feed formulation.

## Introduction

Interest in insect farming is steadily growing in Europe, as insects represent a sustainable protein source [1–3], particularly in response to the rising global demand for protein [4]. Approximately 14% of food produced for human consumption is wasted before reaching retail [5], and globally one-third of food production ends up as waste [6]. Insects such as *Tenebrio molitor* Linnaeus, 1758 (Coleoptera: Tenebrionidae) offer a promising solution for valorising agro-industrial byproducts [7–13] while providing nutritional content comparable to meat [14]. With a protein content of 40–60% on a dry matter basis [15–17], *T. molitor* serves as a valuable source of essentials amino acid, though it contains lower levels of tryptophan and lysine [16–18]. Furthermore, it provides significant amounts of minerals and vitamins [17–19].

Beyond its nutritional benefits, *T. molitor* farming has a markedly lower environmental impact compared to conventional livestock farming. For instance, the direct CO_2_ equivalent emissions per kilogram of mass gain from *T. molitor* are ten times lower than those from pig farming and only one percent of those from beef farming [20]. According to the life cycle assessment conducted by Oonincx and De Boer in 2012, mealworm farming has a lower feed conversion ratio than conventional livestock farming, similar to that of chicken production, and requires less land, though it has higher energy consumption [2]. Consequently, the global warming potential per kilogram of edible protein in mealworm is 1.3 to 3.9 times lower than that of chicken and pork and 5.5 to 12.5 times lower than that of beef [2]. The same study highlights that 56% of this global warming potential stems from the production and transport of mealworm feed. Typical diets in *T. molitor* farming, such as carrots as a wet feed source, contribute 14% to this impact, while mixed grains, as a dry feed source, contribute 42% [2]. Optimising mealworm feed is therefore a key lever for further reducing the environmental footprint of mealworm farming, reinforcing its potential as an alternative to conventional livestock farming. Expanding our understanding of potential insect food sources requires better knowledge of their nutritional requirements and the identification of potentially toxic compounds. Addressing the latter concern is the purpose of this study.

In this context, former foodstuffs from the food industry – such as those discarded due to manufacturing defects (e.g., recipe errors, final shape, cooking) or packaging issues (e.g., mislabeled or improperly packed products) – offer a promising alternative to conventional animal feed [21,22]. Such former foodstuff can account for up to four percent of total production in a confectionery factory, two percent in the chocolate product category, and seven percent in the chocolate biscuit category [23]. These byproducts are already partially used as alternative feed ingredients, mainly for pigs and, to a lesser extent, ruminants [24]. In insect farming, *T. molitor* has demonstrated the ability to feed on a variety of former foodstuffs and industrial byproducts [7–13]. However, while such byproducts present a promising alternative feed source, certain components that are safe for human consumption may be harmful to insects. A question arises with chocolate, which contains theobromine. Theobromine is a methylxanthine compound belonging to the alkaloid class, a group of chemical compounds that may be associated with insecticidal effects [25,26].

Methylxanthines such as theophylline and caffeine have been shown to cause various adverse reactions in insects such as larvae of the moth *Manduca sexta* (Linnaeus, 1763) (Lepidoptera: Sphingidae), including anti-feeding effect, hyperactivity, tremors, and stunted growth, effectively acting as natural insecticides [26]. Studies on hornet *Vespa orientalis* Linnaeus, 1771 (Hymenoptera: Vespidae) revealed that low doses of theobromine (5–10 µg/day) increased appetite for proteins, mobility, and sensitivity to stimuli without affecting mortality. In contrast, prolonged ingestion of higher doses of caffeine (20 µg/day) led to mortality [27]. In *T. molitor*, 3-isobuthylmethylxanthine (a synthetic methylxanthine) has been shown to inhibit food consumption, with an effective dose ranging from 1000 to 3000 mg/kg of feed [26], while pure undiluted theobromine powder (98%), induced spasmodic movements, followed by immobility after several days [28].

Given these observations, it is reasonable to hypothesise that theobromine may impact the development of *T. molitor.* However, current knowledge remains insufficient to clearly characterise its effect on key production parameters, such as survival rate and larval growth. This study aims to determine whether theobromine influences the growth and survival rate of *T. molitor* larvae, and if an effect is observed, to elucidate its nature and magnitude. Additionally, the larvae’s tolerance threshold to varying concentrations of theobromine without incurring adverse effects will be assessed, ultimately enabling the use of chocolate- containing byproducts and former foodstuffs as sustainable feed source for insect cultivation.

## Materials and methods

### Insects

Larvae of *Tenebrio molitor* used in this study were provided by Inagro Insect Research Centre (Rumbeke-Beitem, Belgium) where they were fed *ad libitum* with Insectus (Mijten, Bekkevoort, Belgium) as a dry feed and agar cube as a wet feed. The larvae were around four weeks old after oviposition with a mean individual weight of 7.08 ± 1.21 mg (standard deviation) at the beginning of the experiment.

### Diet preparation

Wheat bran was purchased from ADM Bazancourt (Bazancourt, France) and sieved using a Haver EML digital plus test sieve shaker (VWR, United States) to separate particles inferior to 500 µm (referred to as wheat flour) from those between 500 µm and 1 mm (defined as wheat bran), while removing particles larger than 1 mm. To ensure the controlled addition of theobromine, wheat flour was mixed with pure theobromine (99%, *Thermo Scientific Chemicals*) in a glass beaker to obtain a spiked flour of 18.00 g/kg of theobromine.

Various proportions of wheat flour and spiked flour were mixed to prepare 60.00 g batches of mixed flour. These were then combined with 120.00 g of wheat bran to create substrates with theobromine concentrations ranging from 0 mg/kg (control group) to 5000 mg/kg, as detailed in Table 1. This ratio was chosen to approximate the natural composition of wheat bran. Approximately 50 g of each batch was sent for theobromine analysis (AGROLAB LUFA GmbH, Kiel, Germany). The analysis followed the standard procedure §64 LFGB L 18.00-16:1999-11 (mod.), a modified version of the official German method for the determination of theobromine and caffeine in fine bakery products. The reported uncertainty was ± 10%. These results confirmed that theobromine concentrations in the mixed substrates were within the desired range, validating their preparation (see Table 1). The substrates were stored in the dark at room temperature until the beginning of the experiment.

**Table 1:** Calculated and analysed theobromine levels of different substrates.

Calculated theobromine level (mg/kg)	Analysed theobromine level ± 10% (mg/kg)
0	<10.0
200	207
400	375
600	575
800	750
1000	938
1200	1140
1400	1350
1600	1530
2000	1900
2500	2380
3000	2810
5000	4750

### Rearing conditions

Larvae were reared in a temperature-controlled container equipped with the SCI-40-AV+ refrigeration unit (Maersk Container Industry, Tinglev, Denmark), maintained at 26 ± 1.5 °C and 70 ± 20% relative humidity. The larvae were always kept in the dark except when providing water and during weekly measurements. The entire experiment lasted 10 weeks.

For each substrate, 6 replicates containing 50 larvae each were created. Each batch of 50 larvae was placed in a 15 × 10 × 6 cm plastic container with aeration holes on the sides. Each container was supplied with an excess amount of dry diet (15 g initially) and water was provided *ad libitum* using agar gel (20 g/L of agar powder, VWR Chemicals BDH, Leuven, Belgium), which was supplied 2–3 times a week. If fungi appeared on the agar cubes, they were removed, avoiding the loss of substrate that might be stuck to the agar. The pupae were removed and counted during the weekly measurements.

### Individual weight and mortality rate measurement

Larvae were counted and weighed as a batch each week until 10% of the larvae in the six replicates of each condition had reached pupation. To calculate the number of dead larvae per week, the initial number of larvae was reduced by the number of alive larvae and those that had pupated by that week. For batches where the total number of larvae at week one differed from that at week zero, the count at week one was used as the initial number of larvae.

### Statistical analysis

Statistical analyses were performed using R statistical software version 4.4.0 https://www.r-project.org/ (R Foundation, Vienna, Austria). The effect of concentration of theobromine on the time to 10% pupation was assessed using a Kruskal-Wallis test, as the homoscedasticity assumption for a parametric test was not fulfilled. For post hoc comparisons, a Dunn’s test with a Bonferroni correction was carried out to adjust for multiple comparisons. A p-value lower than 0.05 was considered statistically significant.

Mean individual larval weights (ILW) as a function of time and theobromine exposure were analysed using repeated measures ANOVA. To address the assumption of homoscedasticity, a square root transformation was applied, and the Greenhouse-Geisser correction was used for violations of sphericity. One replicate was excluded from the analysis due to a missing value in a specific week. Normality of the transformed data set was assessed using the Shapiro-Wilk test for each combination of week and theobromine exposure (p-values > 0.05). To evaluate significant differences in ILW by theobromine exposure levels within each week, marginal estimates were used to obtain adjusted means. Post hoc tests were performed using pairwise comparisons of the estimated marginal means, with Bonferroni correction to adjust for multiple comparisons.

To analyse the effect of theobromine quantity on ILW at the week of the first 10% pupation, a Generalised Least Squares (GLS) model was employed. Autocorrelation of the residuals was initially assessed using the Durbin-Watson test on an Ordinary Least Squares (OLS) model, revealing significant first-order autocorrelation. Consequently, the GLS model was adjusted to account for first-order autoregressive correlation of the residuals. The model can be expressed as follows:


$$ILWi\;=\;\beta_{0}\;+\;\beta_{1}\;*\;Theobromine\;+\;\epsilon_{i}\mathrm{\backslash ]}$$

Where ILW represents individual larval weight (mg), Theobromine refers to the theobromine quantity in the substrate (mg/kg), β_0_ is the intercept, β_1_ the regression coefficient and ε_i_ follows a first-order autoregressive correlation structure within the level of Theobromine.

Since a traditional *R*^2^ is not applicable to GLS models, a pseudo-*R*^2^ was calculated by comparing the residual sum of squares (RSS) from the null model (which includes only an intercept) to the residual sum of squares from the full model. The pseudo-*R*^2^ was derived using the following formula:


$$R^{2}=1-\;\frac{{RSS}_{full\;model}}{{RSS}_{null\;model}}$$

Considering the mortality rate, the ANOVA assumptions were not met, even after data transformation, and no suitable parametric model could be identified. Therefore, a non-parametric approach was applied to analyse the data, using the Kruskal-Wallis test for each week. This method was chosen as it does not require the assumptions of normality or homoscedasticity, which were violated in the data. For post hoc comparisons, a Dunn’s test with a Bonferroni correction was carried out with a p-value lower than 0.05 considered as statistically significant.

## Results and discussion

### Larval growth

**Fig 1:**
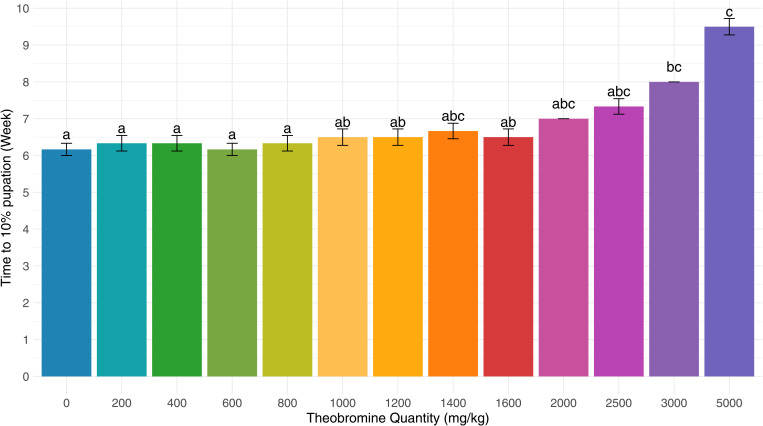
The time (in weeks from the start of the experiment) to reach 10% pupation. Error bars indicate the standard error of the means. Different letters denote a significant difference (Dunn‘s test with Bonferroni correction, p-value < 0.05).

The time to reach 10% pupation varied significantly across theobromine concentrations (Fig 1, p-value <0.001). Larvae exposed to at most 2500 mg/kg reached 10% pupation within 6.5 ± 0.6 weeks (no significant differences to the control), while those exposed to 5000 mg/kg required up to 10 weeks (9.5 ± 0.5 weeks; p-value <0.001). Concentrations of at least 3000 mg/kg delayed pupation by almost 2 weeks compared to the control (p-value = 0.006 for 3000 mg/kg), suggesting a threshold effect around this value.

Nevertheless, the observed threshold is likely to be overestimated, as more frequent measurements, such as daily monitoring instead of weekly, could reveal a more detailed onset of the impact. The observable impact of the methylxanthine theobromine on *T. molitor* pupation aligns with the results of Maguire [29], which demonstrates a delay in the timing and rate of pupation for larvae of *Galleria mellonella* (Linnaeus, 1758) (Lepidoptera: Pyralidae) force-fed with 20 μl of a caffeine solution (0.1 M). Though, under those conditions the larvae were exposed to a methylxanthine at the end of their life cycle, and thus the impact on larval weight was not studied. In contrast, in the present study, theobromine consumption is chronic from the start of growth and this delay could be linked to a reduced larval weight and, consequently a longer growth time. A point that will be explored further in the following paragraphs.

**Fig 2:**
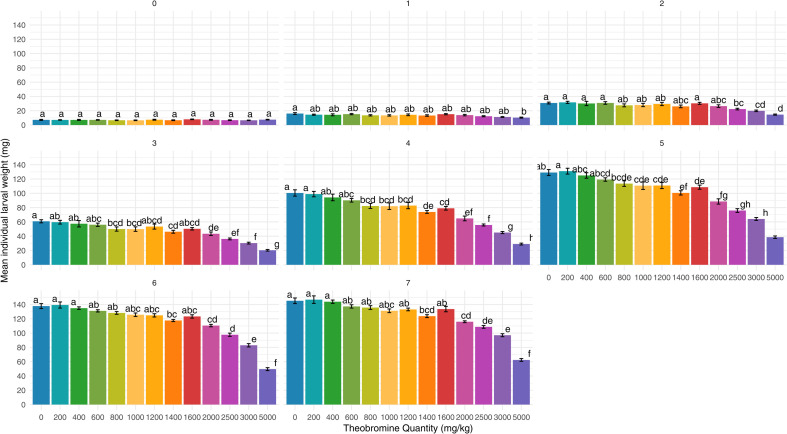
Mean individual larval weights at different feed’s theobromine concentrations (in mg/kg) over 7 weeks. Error bars represent the standard error of the mean. Different letters denote a significant difference (pairwise comparisons with Bonferroni correction, p-value < 0.05).

The effect of the interaction between theobromine exposure and time is significant when considering the square-root-transformed ILW (p-value < 0.001). The mean ILW per week and theobromine concentration is illustrated in Fig 2. Significant differences in the square-root-transformed mean ILW were already seen in the first week between the control and the 5000 mg/kg dosage with respectively a mean weight of 16.0 ± 3.2 mg versus 10.4 ± 1.7 mg (p-value = 0.025). By week 4, significant differences with the control occurred at dosages strictly above 600 mg/kg (p-value < 0.001 except for 800 and 1200 mg/kg, respectively p-value = 0.001 and p-value = 0.002). But interestingly, these differences disappeared later on as by week 6 and 7 there were no significant differences anymore between the control and dosages equal to or below 1200 mg/kg (p-value ≥ 0.434, 132.6 ± 7.7 mg week 6, 139.8 ± 9.2 mg week 7). During these two weeks it should be noted that larvae exposed to 1400 mg/kg showed a significantly lower weight (117.6 ± 3.6 mg, p-value = 0.031 at week 6; 123.7 ± 5.0 mg, p-value = 0.002 at week 7), whereas no significant difference was observed to the higher dose of 1600 mg/kg (123.3 ± 5.7 mg, p-value = 0.241 at week 6; 133.6 ± 9.2 mg, p-value = 1.000 week 7) compared to the control (137.8 ± 8.5 mg week 6, 145.2 ± 9.6 mg week 7). An exposure to 2000 mg/kg of theobromine led to a reduction of around 20% in ILW compared to the control when larvae reached maturity (115.9 ± 3.1 mg, p-value < 0.001). Overall, larvae exposed to higher levels of theobromine tended to have lower mean individual weights.

The fact that more conditions showed significant differences from the control in the first five weeks compared to the following weeks can likely be attributed to the different growth stages of the larvae. Considering the sigmoidal growth pattern of *T. molitor* [30,31], by week 5, the control larvae have likely reached the end of their exponential phase, or even the beginning of their transitional phase, while the larvae exposed to higher concentration (≥ 800 mg/kg) were still in the exponential phase which lead to smaller differences the following weeks. This suggests that while higher doses of theobromine might initially delay growth, the larvae eventually continue to grow, narrowing the gap over time. No significant differences were observed at any time point for larvae exposed to lower concentrations (0–600 mg/kg), suggesting that these doses did not induce detectable growth differences.

Usually, larvae are harvested when the first pupae become visible [7,12,32,33]. Using the results from week 7 as a reference – when most conditions reached 10% pupation – or from week 6 – when first pupae were visible in all batches for each theobromine concentration from 0 to 1600 mg/kg –, no significant impact of theobromine up to 1200 mg/kg was observed on larval weight. However, given the decrease in growth and protein content in the last larval instars, harvesting at earlier instars would be more economically and nutritionally advantageous [33,34]. Based on this, the theobromine concentration threshold for a significant impact on harvest weight appears to be around 600 mg/kg.

The effect of theobromine on larval growth may result from the anti-feeding properties of methylxanthines, as observed previously in *Manduca sexta* and *T. molitor* [26]. This effect can be generally attributed to the alkaloid class (including caffeine), as observed in other insects such as the blow fly *Phormia regina* Rondani, 1862 (Diptera: Calliphoridae) or the moth *Lymantria dispar* (Linnaeus, 1758) (Lepidoptera: Erebidae) [35,36]. This could explain why re-feeding was not required for the higher-exposure batches to guarantee excess feed availability during the experiment, whereas it seemed necessary for the lower-exposure ones.

It could also be hypothesised that larvae had an aversion to the spiked feed. The potential repellent effect of methylxanthines on *Tenebrio molitor* larvae was investigated by Kobetičová (2019), and while an avoidance response to caffeine was demonstrated, no such effect was observed with theobromine [28]. These tests were, however, conducted under different conditions, using aqueous solutions on paper over short periods (96h).

For batches with concentration above or equal to 2000 mg/kg, dry feed was refreshed in week 4 with 2 g of substrate to ensure the feed was provided in excess. Visually, more uneaten food was observed at the highest theobromine levels, although no measurements were taken to confirm this observation or to estimate the associated feed conversion ratio (ratio of feed intake to weight gain). Further research could focus on the impact of theobromine on the feed conversion ratio, as most studies currently investigated the effects of methylxanthine on insects not intended for farming [26,29,37], which leaves a gap in knowledge regarding rearing application. Studies measuring theobromine ingestion in larvae could support these tests by verifying a potential repellent effect of theobromine.

**Fig 3:**
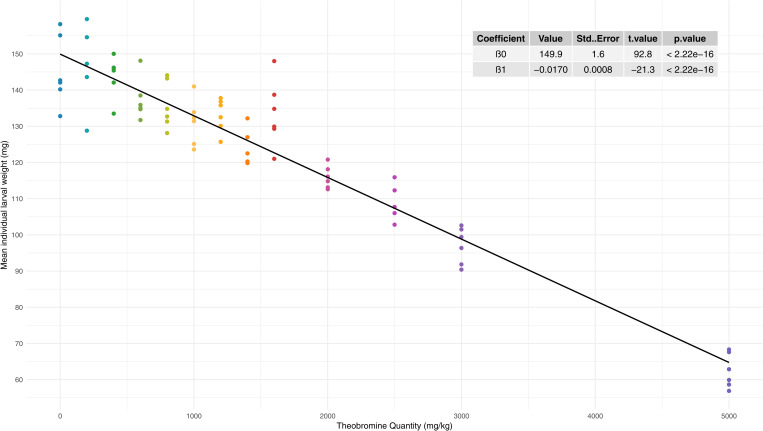
Mean individual weight in function of theobromine dosage at week 7 with the associated GLS representation, pseudo-*R*^2^ = 0.91.

Rather than modelling weight as a function of both time and theobromine concentration, the analysis was focused on a specific time point to ensure robust comparisons. Week 7 was selected as it corresponds to the time when 10% pupation was observed in the control and most conditions. Fig 3 details the GLS model established for this week, which demonstrated a satisfactory fit, as indicated by the pseudo-*R*^2^ of 0.91.

At week 7, a significant linear relationship was observed, indicating a negative impact of theobromine concentration in the substrate on larval weight.

To complete this analysis, Table 2 represents the final weights measured when 10% of the pupae were observed for each replicate with their respective standard deviations. These values are reported for exploratory purposes, as no statistical model could be identified as relevant for a robust analysis considering the time differences between conditions.

**Table 2:** Summary of final weight at 10% pupation and time to reach it, depending on the theobromine concentration.

Theobromine Quantity (mg/kg)	Mean final weight (mg)	Standard Deviation final weight	Time to 10% of pupation (weeks)	Standard deviation of time to 10% of pupation
0	139.2	6.3	6.2	0.4
200	139.7	8.9	6.2	0.4
400	138.2	4.2	6.3	0.5
600	131.4	3.7	6.2	0.4
800	131.8	7.2	6.3	0.5
1000	129.2	6.9	6.5	0.5
1200	129.3	3.7	6.5	0.5
1400	121.5	3.8	6.7	0.5
1600	129.1	5.1	6.5	0.5
2000	115.9	3.1	7.0	0.0
2500	110.4	3.4	7.3	0.5
3000	104.1	4.0	8.0	0.0
5000	82.5	6.7	9.5	0.5

### Mortality rate

**Fig 4:**
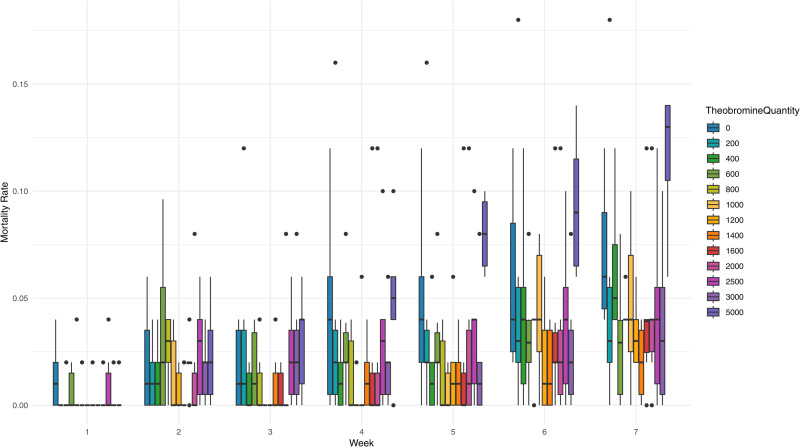
Box plot of mortality rate across weeks at different theobromine levels.

Across all conditions, the average mortality rate was 5% ± 4% at week 7, when most of the group reached 10% pupation. This aligns with the literature on a wheat bran based feed, which reports mortality rate between 0 and 12% under similar conditions [7,38,39] or around 11% on an industrial scale [40]. The Kruskal-Wallis test detected a significant overall difference at week 5 (p-value = 0.046), and post hoc comparisons revealed a difference only between larvae exposed to 1000 and 5000 mg/kg, respectively the lowest mortality rate (1 ± 1%) and the highest (8 ± 2%, p-value = 0.018). At week 7, a significant overall difference was also observed (p-value = 0.016), and this time post hoc tests indicated a higher mortality rate in larvae exposed to 5000 mg/kg of theobromine (12 ± 3%) compared to those exposed to 600 mg/kg (3 ± 3%, p-value = 0.023) and 1400 mg/kg (2 ± 2%, p-value = 0.005). The lack of other significant differences in post hoc tests may be due to the high variability in survival rates, as illustrated in Fig 4. A mortality rate of 18 ± 8% was recorded at week 10 for larvae exposed to 5000 mg/kg, higher than at week 7. However, the time gap complicates the interpretation of this result. This mortality rate can be compared to those observed with other non-optimised diets based on food byproducts where longer growth time is observable with higher mortality rates at the growth end [7,39,41]. A phenomenon also observable when physical conditions are not optimised [30].

These results suggest that if theobromine affects larval mortality, it is likely linked to chronic ingestion over multiple weeks, with a potential threshold above 3000 mg/kg. Alternatively, mortality might result indirectly from feed avoidance or theobromine’s antifeedant properties, leading to larval starvation. However, due to variability in the data and uncertainty about actual theobromine intake, no definitive conclusion can be drawn regarding the threshold or the underlying mechanism. Nevertheless, this potential threshold approaches the theobromine levels found in sweet chocolate (around 4600 mg/kg) [42].

To summarise, this study demonstrated that the presence of theobromine in *T. molitor’*s feed up to 2500 mg/kg had no significant effect on the time required to reach 10% pupation, and up to 3000 mg/kg, no impact on mortality rate was observed. Concentrations up to 1600 mg/kg had no significant effect on final weight, time to reach it, or mortality rates. This aligns with the levels of theobromine found in milk chocolate (approximately 1500 mg/kg), which is about three times lower than the amount in sweet chocolate or four to five times lower than the amount in dark chocolate [42,43] and comparable to levels found in bakery products as brownies, chocolate cake and cookies (1100–1400 mg/kg) [44]. Finally, theobromine levels up to 600 mg/kg did not show a significant effect on overall development compared to no exposure. These findings suggest that *T. molitor* could potentially consume chocolate-related foodstuffs as part of its diet with regard to theobromine content.

Further studies on the impact of theobromine on *T. molitor* larvae are warranted. Specifically, assessing the feed conversion ratio could help confirm and quantify the antifeedant or deterrence properties of theobromine in *T. molitor*. Measuring the amount of theobromine ingested should support such a study. Trials with byproducts and former foodstuffs containing chocolate are expected. The nutritional aspect comes into play (chocolate alone may be too rich in fat for the larvae) [45,46], and theobromine, which is naturally present, could act differently in its matrix. The presence of low levels of caffeine in chocolate products [42,44] could also have an impact on larval growth. An effect of caffeine alone could be expected as a synergistic effect between these two methylxanthines. Additionally, investigating the full growth cycle of the insect, extending to the adult stage, the egg laying, the pupation duration or even spanning multiple generations, could more precisely evaluate a toxic threshold of theobromine in *T. molitor*.

Lastly, examining its potential effects on the nutritional composition of the larvae, particularly regarding the possible residues of theobromine, could provide valuable insights to complement this study for the use of mealworms fed with chocolate-related foodstuff in feed and food. Indeed, mealworms are increasingly used as alternative ingredients in pet food for cats and dogs, and exotic pets such as reptiles and birds. They are also incorporated into livestock and aquaculture feed, including that for broiler chickens and fish [47–51]. Theobromine has shown toxic effects in cats and dogs, may influence feed consumption and conversion ratio in chickens, and has been associated with reduced weight in fish [52,53]. A further consideration is the regulatory framework surrounding theobromine in animal feed. For example, under European legislation, the maximum permissible level in a complete feedstuff is 300 mg/kg, with stricter limits applying to specific species [54]. Such constraints highlight the importance of assessing theobromine metabolism and potential accumulation in *T. molitor* larvae, in order to evaluate their compliance and suitability as feed ingredients.

## Conclusion

The presence of low concentrations of theobromine in *T. molitor* feed does not significantly affect larval growth or mortality (up to 600 mg/kg). However, a dose-dependent relationship was observed, with higher concentration leading to reduced growth. No conclusive evidence was found to suggest that theobromine exposure impact larval mortality except at the highest tested dose (5000 mg/kg). These findings suggest that *T. molitor* larvae can tolerate moderate theobromine levels commonly found in foodstuffs, but excessive concentrations may hinder development. Further research should explore the long-term effects of theobromine, including its potential influence on pupation dynamics, adult emergence, and reproductive success. Additionally, assessing its antifeedant or deterrence properties and its impact on the nutritional composition of larvae (including the theobromine residues in mealworms) could provide valuable insights into its role in *T. molitor diets*. Finally, testing the effect of chocolate as a theobromine-containing matrix would complete this study by evaluating the compound in a realistic dietary context.
